# Ultrathin Solid Polymer Electrolyte Design for High‐Performance Li Metal Batteries: A Perspective of Synthetic Chemistry

**DOI:** 10.1002/advs.202205233

**Published:** 2022-11-28

**Authors:** Qian Wang, Shi Wang, Tiantian Lu, Lixiang Guan, Lifeng Hou, Huayun Du, Huan Wei, Xiaoda Liu, Yinghui Wei, Henghui Zhou

**Affiliations:** ^1^ College of Materials Science and Engineering Taiyuan University of Technology Taiyuan Shanxi 030024 China; ^2^ College of Chemistry and Molecular Engineering Peking University Beijing 100871 China; ^3^ Corrosion and Protection Engineering Technology Research Center of Shanxi Province Taiyuan Shanxi 030024 China; ^4^ State Key Laboratory of Organic Electronics and Information Displays (SKLOEID) Institute of Advanced Materials (IAM) Nanjing University of Posts & Telecommunications Nanjing 210023 China

**Keywords:** energy density, safety, solid polymer electrolyte, synthetic chemistry, ultra‐thin

## Abstract

Li metal batteries (LMBs) have attracted widespread attention in recent years because of their high energy densities. But traditional LMBs using liquid electrolyte have potential safety hazards, such as: leakage and flammability. Replacing liquid electrolyte with solid polymer electrolyte (SPE) can not only significantly improve the safety, but also improve the energy density of LMBs. However, till now, there is only limited success in improving the various physical and chemical properties of SPE, especially in thickness, posing great obstacles to further promoting its fundamental and applied studies. In this review, the authors mainly focus on evaluating the merits of ultrathin SPE and summarizing its existing challenges as well as fundamental requirements for designing and manufacturing advanced ultrathin SPE in the future. Meanwhile, the authors outline existing cases related to this field as much as possible and summarize them from the perspective of synthetic chemistry, hoping to provide a comprehensive understanding and serve as a strategic guidance for designing and fabricating high‐performance ultrathin SPE. Challenges and opportunities regarding this burgeoning field are also critically evaluated at the end of this review.

## Graphical abstract

This study mainly focuses on the trend of applying established synthetic chemistries in manipulating the thickness of SPE to bridge its incorporations with practical electrochemical devices with high‐energy‐density LMBs. The advantages and existing challenges of ultrathin SPE are discussed, while the progress of ultrathin SPE based on synthetic chemistry are summarized and several typical fabrication methods are looked back upon.

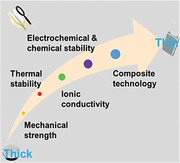

## 1 Introduction

At present, the demand for energy grows with each passing day along with the continuous development and transformation of industry.^[^ ^1^ ^]^ However, traditional fossil fuels (such as: coal, oil, and gas) are going to be used up in this century, and the environmental problems caused by them will become more and more serious.^[^ ^1^, ^2^ ^]^ Against this background, it is imperative to develop clean and efficient new energy represented by solar energy, wind energy, geothermal energy, etc.^[^ ^1^, ^3^ ^]^ Among them, developing electrochemical energy storage devices with high specific energy is of great strategic significance for adjusting the energy structure and improving energy efficiency.^[^ ^1^, ^2^, ^4^ ^]^ As a typical electrochemical energy storage device, lithium‐ions batteries (LIBs) have dominated portable devices, consumer electronics, electric vehicles, and grid energy storage system for decades.^[^ ^5^ ^]^ However, the existing LIBs based on graphite anode (such as: LiFePO_4_|graphite battery) are approaching their energy density ceilings (300 Wh kg^−1^), unable to satisfy the ever growing demand for high energy density, especially in the electric vehicle market.^[^ ^6^ ^]^ Among various newly developed LIBs technologies, the battery systems based on Li metal anode, such as: Li–S batteries, Li–air/oxygen batteries, etc., could deliver a high specific energy of >600 Wh kg^−1^.^[^ ^7^ ^]^ Even if only using Li metal anode instead of current graphite anode and pairing with commercial transition metal oxide cathodes (LiCoO_2_, LiNi*_x_* Co*_y_* Mn_1−_ *_x_*_−_ *_y_* O_2_, LiMn_2_O_4_, etc.), the obtained batteries can also exhibit an energy density of >400 Wh kg^−1^.^[^ ^8^ ^]^

Unfortunately, due to its high reactivity, Li metal will inevitably react with almost all liquid electrolytes to form an unstable solid electrolyte interphase (SEI) film on its surface, causing irreversible capacity decline. Simultaneously, during the subsequent charging/discharging cycles, the bulk phase of Li metal electrode will undergo drastic volume changes because of its matrix‐free feature, exposing a lot of cracks caused by the rupture of fragile SEI film.^[^ ^7^, ^9^ ^]^ Cracks cause uneven current density distribution and enhance the local Li^+^‐flux, triggering uneven Li deposition, thereby leading to notorious Li dendrite growth. Continuous dendrite growth and recurring SEI formation will bring continuous interface side reactions, which seriously reduces the coulombic efficiency (CE) and cycle life, even causes a series of safety hazards, such as: battery short circuit, thermal runaway, explosion, etc. (**Table** **1**).^[^ ^8^, ^10^ ^]^ Besides, commercialized LIBs use organic liquid electrolytes, such as: carbonated solvents (containing lithium salts), which are flammable and volatile, and have the potential to catch on fire or even explode, especially at high temperatures.^[^ ^11^ ^]^ At the same time, liquid electrolytes have higher requirements for the structural design and packaging level of LIBs, leading to fixed housing, high cost, et al., thus it is extremely difficult to realize miniaturized battery products with high‐density integration.^[^ ^12^, ^13^ ^]^ Hence, it is urgently desired to develop a new type of safe electrolyte for the practical application of LMBs.

**Table 1:** Challenge and influence of Li metal anode in traditional LIBs

Challenges	Consequence
Uneven deposition, pores, and pulverization	Capacity decline, volume change, short circuit, and thermal runaway
Side reaction with electrolyte to form SEI	Increase of internal resistance, capacity decline, inflation, and deactivation
Volume expansion and contraction	Poor interface contact, increasing internal resistance, and reducing cycle life
Reaction occurs when exposed to air	Difficult storage, uneven current density, and side reactions
Poor mechanical strength	Difficulties in processing and mass production
Low melting point	Unsafe at high temperature (180 °C)

Based on the background and challenges mentioned above, using solid polymer electrolyte (SPE) instead of traditional liquid electrolyte has attracted widespread attention.^[^ ^2^, ^14^ ^]^ Its progressiveness lies in:^[^ ^15^ ^]^ a) excellent interface compatibility; b) high security; c) long cycling life and capacity retention; d) high energy density; and e) processability. All these advantages have made SPE a hot topic in next generation LMBs, which have triggered great innovations in many fields including synthetic chemistry, materials engineering, coordination chemistry, electronics and energy.^[^ ^16^, ^17^ ^]^ However, low ionic conductivity, narrow electrochemical window (i.e., poor electrochemical stability at high voltage) and unsatisfactory mechanical strength limit its application on LMBs, especially in high voltage Li metal batteries (HVLMBs).^[^ ^18^ ^]^ And then, to make matters worse, for most SPE, ionic conductivity at room temperature and mechanical strength are often self‐contradictory, namely: SPEs with high mechanical strength usually have low ionic conductivity.^[^ ^17^, ^19^ ^]^ Therefore, how to decouple the ionic conductivity and mechanical strength of SPE is extremely important to promote the practical application of LMBs, which has great scientific significance and research value. Advances in the past decade have witnessed the evolution of this research from simple polyoxyethylene (PEO) molecular to well‐designed and multilevel molecular structures by diverse molecular design, including copolymerization, crosslinking, grafting, organic–inorganic hybrid, polymer alloying, introducing 3D network, etc.^[^ ^20^ ^]^ These strategies can alleviate the contradiction between ionic conductivity and mechanical strength to a certain extent, and thus demonstrated great potential. However, despite that current SPE has developed rapidly and achieved considerable success, its thickness used in many reports has surpassed over 100 µm even 500 µm (much thicker than the current liquid electrolyte + separator), can't meet the requirements for practical application.^[^ ^21^ ^]^ The thickness of SPE mainly affects the interface resistance, ionic migration, and energy density of LMBs. When downsizing SPE into smaller thickness scales, for example, from 100 to 20 µm, SPE has dramatically expanded their application horizons because of the special physicochemical properties under the size effect.^[^ ^11i,k^ ^]^ Specifically: a) the energy density, especially volumetric energy density can be improved significantly, and is suitable for some miniaturized battery products; b) because the ionic conductance is inversely proportional to the thickness of SPE, the interface resistance and charge transfer resistance will be greatly reduced, which will improve the rate performance and power density of LMBs, and it is suitable for some batteries with high power in special environment; c) the cost can be reduced obviously, promoting the commercialization of LMBs; and d) flexibility and extensibility are greatly improved, giving stretchable devices a certain possibility. Despite these benefits, the thin or ultrathin SPE is difficult to give consideration toward its mechanical strength, arising as a huge challenge in safety, and cycling life. In brief, ultrathin SPE synthesis and design show great potential for high‐performance LMBs but still have a certain distance from practical applications. How to prepare an advanced ultrathin SPE is becoming an interdisciplinary science with both challenges and opportunities for researchers to exploit being endless. However, it has not been methodologically discussed and timely summarized.

Herein, it is necessary to organize a critical review regarding the challenges and design principles for ultrathin SPE to provide a comprehensive understanding and serve as a strategic guidance for promoting the applications of LMBs. However, most existing reviews on similar topics are concentrated on summarizing various polymer molecular designs in improving the physicochemical properties of SPE, such as: ionic conductivity, Li^+^ transference number, etc., highlighting the superiority of polymer designs and regulations at molecular level.^[^ ^11^, ^22^ ^]^ Admittedly, these are extremely important for SPE, but neglecting another key issue of SPE in practical application, that is, thickness, especially ultrathin SPE, failing to provide the readers with a comprehensive overview about the nature of the problems and development trend of SPE. Thus, as a remedy for existing reviews that have rarely sketched the thickness of SPE, we mainly focused on the trend of applying established synthetic chemistries in manipulating the thickness of SPE to bridge its incorporations with practical electrochemical devices with high‐energy‐density LMBs. To make the scope topical and succinct, we will start with a fundamental introduction to the SPE and its development tendency. Then, this story will turn to the existential challenges, fundamental requirements, and design philosophies of ultrathin SPE. Subsequently, representative ultrathin SPE are highlighted and the underlying principles of selecting appropriate polymer molecules and synthetic/cross‐linked methods are summarized from a perspective of synthetic chemistry. Future directions regarding the structure–property relations and advanced fabrication/manufacturing of ultrathin SPE will also be discussed. We aim to place a unique view on ultrathin SPE, providing an accessible guidebook for future designs and innovations. We hope that such an elaborately organized and prepared review will benefit the readers interested or ready to engage in SPE fields to create more possibilities.

## 2 Solid Polymer Electrolyte and its Future Trends

SPE is a kind of polymer material with ionic conductivity, which is composed of polar polymers and Li salts in the polymer matrix through Lewis acid–base reaction.^[^ ^17^, ^23^ ^]^ Because of its high safety, high mechanical flexibility, viscoelasticity and film‐forming properties, it is considered to be one of the most potential electrolytes in next generation LMBs. Actually, as a lithium ionophore, the Li^+^ migration in the polymers is realized through the wriggle of polymer chains, thus the Li^+^ transportation in the polymer can only be reflected above its vitrification point.^[^ ^24^ ^]^ By that time the polymer chains can move freely, and the Li^+^ migration in the polymers shows a diffusion behavior similar to that in liquid electrolyte.

### 2.1 Merits and Applications

#### 2.1.1 Merits

Driven by the safety hazards of traditional LIBs and the applications of LIBs in complex and harsh environments as well as the emergence of polymer materials, SPE has attracted global attention.^[^ ^11^, ^22^ ^]^ Its main advantages are listed below: a) safety. Compared to liquid electrolytes, SPE can suppress dendritic Li growth efficiently, not easy to burn and explore, no risk of liquid leakage, no side reactions at high temperature, etc., means: LIBs using SPE can work under high rate, thus, LMBs with LiNi*_x_* Co*_y_* Mn_1−_ *_x_*_−_ *_y_* O_2_ cathode will no longer be demonized; b) energy density. In traditional LIBs based on liquid electrolytes, fearing that the side reaction between cathode materials and electrolytes under high voltage, causing irreversible phase transition of cathode materials, some high‐voltage cathode materials are rarely used. However, because of high electrochemical windows, SPE can match with high‐voltage cathode materials to assemble high voltage Li metal batteries (HVLMBs), improving the energy density significantly. Moreover, due to the improved safety, solid‐state batteries can be made more compact, making into a battery pack with only a few or even one unit, thereby showing to be even more advanced than the popular blade batteries; c) rate performance. Different from the LMBs with liquid electrolytes, where the Li metal anode has poor compatibility with electrolyte, especially at a high rate, whereas the solid‐state LMBs can fully realize fast charging and discharging in 10 min without interface side reactions caused by dendritic Li growth when the ionic conductivity of SPE is high enough; d) low temperature characteristics. Liquid LMBs will display serious polarization and capacity decline at low temperature because of the mismatch of electron migration speed between internal and external circuits of battery, causing the increased electrolyte viscosity and slower Li^+^ transport. However, after using SPE instead of liquid electrolyte, the LMBs will not freeze as the liquid battery with the cooling, which will lead to the failure of battery operation, and their safety is high; e) cycling life. Using SPE can effectively avoid the formation of SEI film on the surface of Li metal anode, improving the utilization rate of Li metal anode and cycling life; f) light weight. Solid‐state LMBs do not require electrolyte and separator, which can simplify the packaging and cooling system, thereby reducing the weight and volume of the overall battery pack; and g) battery management system (BMS). Solid‐state LMBs can operate in a wide temperature range, thus the demand for BMS will be reduced accordingly.

**Figure 1:**
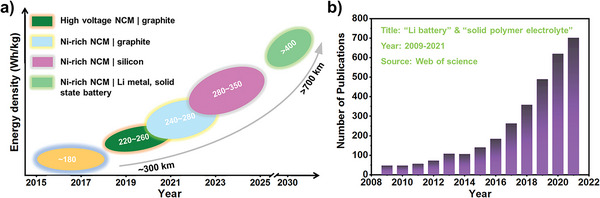
Current status and future trends of solid polymer electrolyte (SPE). a) Technical route and development trend of power battery. b) Publications about SPE from 2009 to 2021.

All in all, SPE behaviors have distinctive features in energy density, safety, BMS, etc. which brings a very bright application prospect. Meanwhile, from the technical development route of power battery, solid‐state LMBs demonstrate attractive prospects in terms of energy density, and they will be mass produced around 2030 according to reasonable speculation (**Figure** **1**a). When combining SPE with Li metal anode and LiNi*_x_* Co*_y_* Mn_1−_ *_x_*_−_ *_y_* O_2_ cathode, the obtained batteries can exhibit an energy density of >400 Wh kg^−1^, means: an electric vehicle would travel 700 km after a single full charge, overcoming petrol vehicles in the future market. It seems an inevitable choice to use solid‐state LMBs in the next‐generation battery systems. In addition, the growing market demands for energy density leads to world‐wide research surge in SPE for next‐generation rechargeable LMBs. The relevant publications also exhibited exponential increase since 2010s (Figure 1b).

#### 2.1.2 Applications

**Figure 2:**
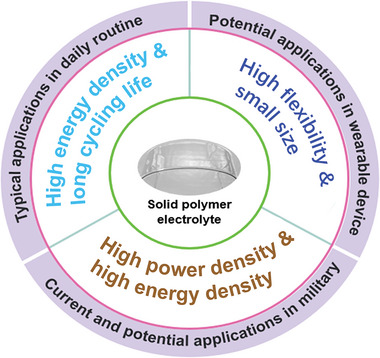
Current and potential applications of Li‐ions batteries with ultrathin SPE in daily routine, military, and wearable devices.

With the continuous progress of SPE design and battery manufacturing, the application scope of solid‐state LMBs with SPE will be expanding, and its current and potential applications are as follows (**Figure** **2**):

#### 2.1.3 Applications in Daily Routine

The current applications of LIBs in daily routine mainly focus on electronic products and vehicles. For electronic products, the typical devices mainly including: mobile phones, laptops, digital cameras, mobile DVDs, cameras, MP3 players, etc. Take laptops as an example only, its annual demand for batteries has reached billions of dollars and maintained a continuous growth of >10% every year. Now, portability has become one of the preferred factors for consumers to choose laptops, and as one of the heaviest parts, LIBs will also develop toward a lighter and thinner direction. The traditional LIBs thus will gradually be replaced by solid‐state LMBs being with light weight and large capacity. Obviously, regardless of other aspects, just from this point, SPE will continue to develop toward a thinner and thinner direction. For the applications on vehicles, the typical devices mainly including: electric cars, electric bicycles, electric motorcycles, golf carts, electric wheelchairs, electric (disabled) scooters, electric buses, electric skateboards, and even electric children's amusement vehicles, etc. Among them, as a new form of urban transport, electric cars have a broad development space in the 21st century with the theme of sustainable development and ecological environmental protection because of its advantages of being clean and pollution‐free. However, it is hard for current LMBs to alleviate the driver's psychological shadow about range anxiety, which can be described as “energy hunger.” In that case, solid‐state LMBs with ultrathin SPE can make up for this defect.^[^ ^24^, ^25^ ^]^

#### 2.1.4 Applications in Defense and Aerospace

Defense and aerospace have always been the center of high technology. Various military equipment, especially weapons used in ground operations, cannot be separated from the LIBs with high specific energy, excellent high/low temperature performance, being lightweight, and miniaturization. Meanwhile, the LIBs used in communication and aerospace also tend to be of high safety, of long cycling life, of high specific energy, and lightweight. Take the current main application of LIBs in the aviation field as an example, unmanned small/micro reconnaissance planes require the LIBs to have the characteristics of high reliability, stable low‐temperature performance, high energy density, small volume and mass, thereby reducing the launch cost. Looking at various battery technologies, perhaps only solid‐state LMBs with ultrathin SPE can meet the above requirements.

#### 2.1.5 Applications in Wearable Devices

With the rapid development of wearable technology, flexible wearable LIBs came into being. Only with excellent flexibility, high energy density and stable dynamic power output, flexible wearable LIBs can be used in various flexible devices, such as: flexible displays, wearable devices and smart cards, meantime, it still stably provides the power even in the case of mechanical deformation of the devices. As a new human friendly electronic product, the flexible stretchable device has broad application prospects in the fields of Internet of things, artificial intelligence, health care, and man–machine interactive.^[^ ^23^, ^26^ ^]^ However, as the core functional layer of devices, electrolyte materials lag behind the development. To put it more bluntly, for flexible/stretchable devices, batteries should be designed around electronic devices, not electronic devices around batteries. If ultra‐thin polymer electrolyte is used in flexible/stretchable devices, it is not only a gospel for device strain, but also very important for device volume and energy density. For meeting the needs of flexible stretchable electronic products, such as: wearable electronic devices, bionic prosthetics, soft robots, electronic skin, etc., it is urgent to develop matching stretchable batteries, which has become a bottleneck problem. Ultrathin SPE can not only bend, but also stretch, wind and compress, which is of great significance to solve the above bottlenecks.

### 2.2 Classification According to Synthetic Chemistries

The structure and composition of SPE have an important effect on its physical, chemical, and electrochemical properties. Typical homopolymers (such as: polyethylene oxide, PEO) are usually limited in application because of their single performance and serious defects.^[^ ^11^, ^12^, ^27^ ^]^ Synthesizing the SPE with different structures thus has become one of the commonly used means for researchers to endow SPE with multifunction. After several decades of development, researchers have long been dissatisfied with the exploration of SPE with single structure and function. Up to now, many SPE systems have been developed, according to their structure and composition, we can roughly divide them into the following types from the perspective of synthetic chemistry (**Table** **2**). In this section, we will briefly introduce them in order to explore appropriate construction strategies for ultra‐thin SPE in the subsequent sections.

**Table 2:** Classification of SPE according to synthetic chemistries

Classification	Ionic conductivity	Mechanical strength	Electrochemical stability
All solid polymer electrolyte	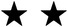	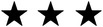	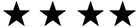
Gel polymer electrolyte	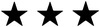	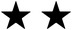	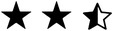
Porous polymer electrolyte	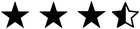	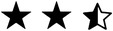	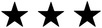
Composite polymer electrolyte	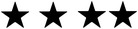	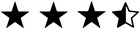	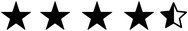

#### 2.2.1 All Solid‐State Polymer Electrolyte

PEO is the earliest and most widely studied SPE. However, its high crystallinity makes it difficult for Li^+^ to migrate at room temperature, researchers thus have developed a series of strategies to reduce the crystallinity of PEO and improve its ionic conductivity.^[^ ^28^ ^]^ Besides, SPE also needs to increase shear modulus (mechanical strength) to meet its application requirements. Common SPE modification strategies mainly include cross‐linking, grafting, block copolymerization, etc. Among them, cross‐linking is the most common method because it can not only increase the shear modulus of SPE, but also avoid the orderly arrangement of polymer chains. Cross‐linking point is the connection point of multiple chain segments, which can be divided into fixed cross‐linking point and sliding cross‐linking point. In addition to PEO, other polymers are also used as SPE, for example, polycarbonate (PC). Due to the strong polar carbonate group in the main chain structure of PC and its amorphous property at room temperature, Li salts are much easier to dissociate in PC, and the corresponding ionic conductivity is higher, it thus is regarded as a potential alternative material for PEO. Besides, other SPE designs, including: block copolymers, graft polymers, star polymers, etc., can balance the mechanical strength and ionic conductivity.

#### 2.2.2 Gel Polymer Electrolyte

As a transitional product before liquid electrolyte and all solid electrolyte, gel polymer electrolyte combines the characteristics of toughness (from SPE) and easy diffusion (from liquid electrolyte), overcomes the shortcomings of liquid leakage and improves the ionic conductivity, lowering interface side reactions, thereby endowing the diversity of electrolyte designs. For example, introducing plasticizers with low molecular weight, such as: organic carbonates into SPE matrix can form a gel polymer electrolyte (GPE). Via this method, the ionic conductivity of GPE can be significantly improved, reaching more than 10^−3^ S cm^−1^ at room temperature, however, adding plasticizers with low molecular weight will greatly reduce the mechanical properties of solid electrolytes, resulting in potential safety hazards.

#### 2.2.3 Porous Polymer Electrolyte

Porous polymer electrolyte means that the polymer matrix has a porous structure, and plasticizers and Li salts exist in the polymer bulk structure. Polymer porous membrane has high porosity, strong liquid retention ability, and certain mechanical strength. It is different from and related to gel polymer electrolyte: 1) their working mechanism is basically identical and performance is similar; 2) compared to GPE, although porous polymer electrolyte membrane has certain mechanical properties, there are also some problems, such as: electrolyte leakage; and 3) the preparation process of porous polymer electrolyte is simple and easy to realize in large‐scale production. It's worth noting that porous structure has a great influence on the liquid absorption and ion transfer. Increasing the porosity and pore size can wrap more liquid electrolytes in the pores, greatly improve the content of carrier ions, which play a leading role in the improvement of ionic conductivity and Li^+^ transference number. Up to now, PVDF‐HFP is the most widely used porous polymer electrolyte.

#### 2.2.4 Composite Polymer Electrolyte

Organic–inorganic composite can not only increase the ionic conductivity of SPE to a certain extent, but also comprehensively improve its interface properties, electrochemical stability and mechanical properties, which has attracted much attention in recent years. It has the following advantages: 1) improve ionic conductivity. Adding inorganic fillers into the polymer matrix can reduce the crystallinity of SPE and enhance its ionic conductivity; 2) integrate the characteristics of each component. For example, improve the mechanical strength, electrochemical window, interface stability (prevent interface side reactions by physically adsorbing trace impurities in SPE), etc. However, the effect of inorganic materials on SPE is closely related to their types, microstructure, particle size, and surface modification.

### 2.3 What Happens to Ultrathin SPE?

Downsizing SPE into a smaller thickness scale, SPE will dramatically expand its physicochemical properties and application horizons primarily because of the advantages of size effect.^[^ ^11i,k^ ^]^

#### 2.3.1 Energy Density

**Figure 3:**
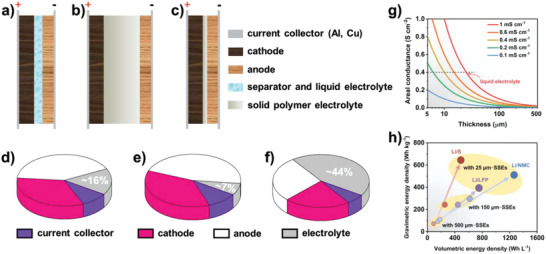
Progressiveness and advantages of ultrathin SPE. a–c) The schematic image of several battery structures with different electrolytes: a) liquid electrolyte; b) thick SPE; and c) ultrathin SPE. d–f) The volume ratio of different electrolytes in Li‐ions battery: d) liquid electrolyte; e) SPE with 100 µm thickness; and f) SPE with 10 µm thickness. g) Relationship between ionic conductivity and the thickness of SPE in Li‐ions battery. Reproduced with permission. Copyright 2021 Royal Society of Chemistry. h) Relationship between gravimetric/volumetric energy densities and the thickness of SPE in Li‐ions battery. Reproduced with permission. Copyright 2021 Royal Society of Chemistry.

When reducing the thickness of SPE, the energy density of LMBs, especially the volume energy density, is significantly affected. According to typical battery structure, the thicknesses of Cu/Al foils (current collector) and separator (separator + liquid electrolyte) are 10 and 25 µm, respectively. It is assumed that the double‐sided density of cathode materials is 20 mg cm^−2^, specific capacity is 140 mAh g^−1^, active materials accounted for 94%; and specific capacity of anode materials is 320 mAh g^−1^, active materials accounted for 94%; safety factor (negative/positive, N/P) is 1.03. Packing density of cathode materials and anode materials can be reached to 3.8 and 1.6 g cm^−3^, respectively. Then, it can be calculated that the thicknesses of cathode plate and anode plate are 50 and 62 µm, respectively. As shown in **Figure** **3**a–d, in traditional LMBs, the volume ratio of liquid electrolyte is around 16%. However, for the thick SPE, such as: 100 µm, its volume ratio will be ≈44%, which is much higher than electrode materials (Figure 3b–f). When the thickness of SPE is reduced to 10 µm (ultrathin), its volume ratio will be reduced to a very small value, only 7% (Figure 3c–e). Obviously, the thickness of SPE plays a decisive role in improving the energy density (especially the volume energy density) of batteries and thinner SPE is essential to achieve higher gravimetric and volumetric energy densities (Figure 3h). However, most SPEs have a thickness >100 µm, even 200 µm, far from meeting the actual demands.

#### 2.3.2 Li^+^ Migration

Apart from energy density, another critical parameter affected by SPE thickness is Li^+^ migration and transport, which depends on charge transfer resistance and ionic conduction of SPE. According to the calculation formula of ionic conductivity, *G* = *σA*/*L*, where *G*, *σ*, *A* and *L* represent the ionic conductance, ionic conductivity, surface area and thickness of the SPE, respectively, it can be seen that the ionic conductance is inversely proportional to the thickness of SPE and simple normalization was performed for convenience. As shown in Figure 3g, higher area‐normalized conductance is observed in solid state electrolytes (SSEs) with reduced thickness, which originates from the shorter time it takes for ions to transport across SSEs (*t* = *L* ^2^/*D*, where *t*, *L* and *D* represent the Li^+^ diffusion time, thickness of solid‐state electrolyte and Li^+^ diffusion constant, respectively).^[^ ^11i,k^ ^]^ To achieve rapid Li^+^ migration and transport, reducing the thickness of SPE is equally important to increasing its ionic conductivity. For example, SPE with ionic conductivity such as 0.4 mS cm^−1^ can obtain the same areal conductance as a liquid electrolyte if the thickness can be reduced to 10 µm.

#### 2.3.3 Flexible/Stretchable Devices

Flexible/stretchable devices are a subversive technology with high cross integration, thus developing soft and elastic batteries like human skin has been promoted as the increasing demand for high‐performance and complex electronic devices, such as: smart bracelets, implantable electronic devices, pacemakers, soft wearable devices, etc. Generally speaking, in reducing the thickness of SPE, the flexibility will be improved, and thus the deformability and stretchability will be greatly increased. Whereas for these characteristics, it is very desirable to accelerate the research and development of ultra‐thin SPE to facilitate the deployment of various portable devices. It is undoubtedly good news for the EVs. Because the design of the car shape will have more freedom and the battery designs can be considered after the design of space and other functional equipment.

## 3 Phylogeny of Ultrathin SPE

In fact, polymer solid electrolyte can be traced back to 1973, when Wright et al. found that the complexes formed by PEO and various alkali metal salts can display certain conductivity. Although this research work did not receive enough attention at that time, because the SPE was formed by dissolving Li salts into polymer, this discovery is usually regarded as the beginning of research on SPE. Subsequently, Armand et al. applied PEO to SPE in 1979, showing great application potential, which started the extensive pursuit of SPE for more than 40 years. In 1983, Berthier et al. pointed out that the high crystallinity of PEO is the main reason for the low conductivity of SPE at room temperature. Based on this understanding, some strategies, such as: cross‐linking, copolymerization, grafting, et al., have been proposed to reduce the crystallinity of PEO. For example, Cheradame et al. obtained a SPE with a high ionic conductivity of 5 × 10^−5^ S cm^−1^ at room temperature by using the synthesis method of cross‐linking and copolymerization. And Boudin et al. prepared a porous PVDF‐HFP electrolyte, which demonstrated a high ionic conductivity of 3.7 × 10^−3^ S cm^−1^ at room temperature. And our group prepared a series of hyperbranched SPEs by using the synthesis method of “active”/controlled radical polymerization, the highest ionic conductivity of the hyperbranched SPE can reach 1.46 × 10^−4^ S cm^−1^ at room temperature.

However, when it comes to the thickness of SPE, it is only in recent years that researchers have paid attention to it. For example, a typical work was reported by Nan Group in 2017, in which they prepared an ultra‐thin SPE (≈100 µm) based on polyvinylidene fluoride/hydroxyethyl cellulose (PVDF/HEC) by a simple dipping method.^[^ ^29^ ^]^ The wide electrochemical window and high Li^+^ conductivity demonstrate that the electrolyte is suitable for LiNi_0.5_Mn_1.5_O_4_ as well as other electrodes. Unfortunately, this thickness of 100 µm is far from meeting the actual needs. Recently, Cui Group reported an interesting ultrathin SPE, and its thickness is only 8 µm.^[^ ^30^ ^]^ Specifically, the SPE used the ultrathin, flexible, mechanically strong, nonflammable, and porous polyimide (PI) film as the host and PEO/LiTFSI as the ionically conducting filler. The obtained hybrid PI/PEO SPE demonstrated cycling stability superior to that of plain PEO in Li/SPE/Li cells under identical current density. Beyond doubt, this work demonstrated great potential of ultra‐thin SPE design in the molecular level to further decouple the ionic conductivity from mechanical properties (thickness, flexibility), offering huge opportunities for modifying the PEO‐based SPE. Nevertheless, we call for more strategies to be developed to synergistically improve the physical and chemical properties of ultrathin SPE.

## 4 Challenges of Ultrathin SPE: Science and Technology

### 4.1 Challenges toward the Ultrathin SPE Itself and its Inherent Properties

#### 4.1.1 Mechanical Strength

**Figure 4:**
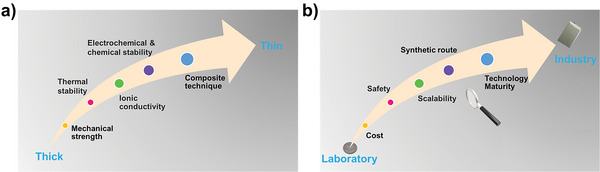
Possible challenges of SPE from thick to thin. a) At the technical level, including: mechanical strength; electrochemical/chemical stability, et al. b) At the manufacturing level, including: cost; safety; technology maturity, et al.

Just as the name implies, it refers to the mechanical response behavior when an external force (such as: dendritic Li growth) applied to the SPE. Generally speaking, the mechanical strength is inversely proportional to its thickness. According to the literature, only when the mechanical strength of SPE is greater than 6 GPa can the dendritic Li growth be effectively suppressed.^[^ ^10^, ^31^ ^]^ Assuming that a SPE (thickness, ≈100 µm) has a mechanical modulus of 6 GPa, when the thickness of SPE is reduced to ≈10 µm (ultrathin), the mechanical strength of the ultrathin SPE must exceed 60 GPa to effectively inhibit the dendritic Li growth (**Figure** **4**a). However, it is extremely challenging for pure polymers, especially PEO.

#### 4.1.2 Stability

There is no doubt that stability has always been one of the most important parameters for SPE, including: thermal stability, electrochemical stability, chemical stability, et al. However, when downsizing SPE into smaller thickness scales, it brings great challenge to the stability of SPE. The first one affected is thermal stability, especially the long‐term thermal stability. It is particularly important for the safety of LMBs, and it can reflect that the LMBs can run normally for a long time in practical applications without deterioration in performance.^[^ ^32^ ^]^ For the ultrathin SPE, once the internal micro short circuit occurs in the LMBs, the local heat can quickly melt the ultrathin SPE in a very short time, leading to fire or even explosion. However, for thick SPE, the local heat is insufficient to melt the SPE, thereby endowing the LMBs with higher safety. Besides, reducing the thickness of SPE is also a great challenge for its electrochemical/chemical stability, which mainly affects the electrode/electrolyte interface. It is widely known that Li metal is very electropositive and reactive, it will spontaneously react with most SPEs during the charging/discharging process. Currently, most typical SPEs have a narrow electrochemical stability window and cannot operate at the full voltage range of the cathode and anode materials. Nonetheless, these SPEs are unstable and can be reduced by Li metal at low voltage, thereby leading to unstable electrode/electrolyte interface and the formation of SEI film. Obviously, for the thick SPE, the poor electrochemical/chemical stability at most leads to unstable electrode/electrolyte interface, which will only lead to the performance degradation of LMBs, but will not cause safety hazards. However, for the ultrathin SPE (such as: 10 µm, even 5 µm), the unstable electrode/electrolyte interface is very likely to cause a sharp drop in the local mechanical properties of ultrathin SPE, resulting in short circuit and even explosion of LMBs (Figure 4a).

#### 4.1.3 Composite Technique

Since the mechanical strength and stability of the ultrathin polymer are difficult to achieve, the future ultrathin SPE is probably based on the strategy of organic–inorganic composite. However, some conventional inorganic materials used for composite are difficult to achieve as ultrathin due to their large particle size, such as: Li_10_GeP_2_S_12_ (LGPS), oxide‐based inorganic materials (LAGP, LLTO, and LATP), which is also a big challenge for ultrathin SPE.^[^ ^32^, ^33^ ^]^ Besides, the research on the compatibility between inorganic fillers and polymer matrix lags behind, especially the research on how to ensure the uniform distribution of inorganic fillers in polymer matrix and the stability during battery run (not “overflow” from SPE). The uniform distribution of inorganic fillers in polymer matrix depends on the accurate targeting between them (Figure 4a). However, most inorganic materials have poor compatibility with polymers, which makes it difficult to effectively composite them with polymer matrix. The current strategies, such as: nano‐sized inorganic fillers, supplemented by long‐time stirring or ultrasound, have not fundamentally improved the compatibility between inorganic fillers and polymer matrix.

### 4.2 Challenges toward Mass Manufacturing

In addition to the above‐mentioned scientific challenges in chemistry and electrochemistry, there is another key technical problem in the practical application for ultrathin SPE, namely, manufacturing, especially mass manufacturing. How to prepare stable and controllable thickness ultrathin SPE on a large scale determines whether it can be applied in actual LMBs (Figure 4b).

#### 4.2.1 Synthetic Route and Cost

Unlike the small‐area ultrathin SPE prepared in the laboratory scale, in industrial production, the first thing to consider is whether the technical route can be realized in mass manufacturing. For some complex and multi‐step polymerization reactions, it is difficult to achieve mass manufacturing in the industrial, which is not only because of its cumbersome process flow, but also because the stability (such as: molecular weight, polymerization degree) of the obtained materials is difficult to maintain after each reaction step. Besides, for the composite ultrathin SPE, it is also a great challenge to realize uniform composite process and ensure the uniform distribution of inorganic fillers in polymer matrix, thus ensuring the stable and rapid Li^+^ transfer, especially the Li^+^ transport path at the two‐phase interface after organic–inorganic composite. However, in the large‐scale production, it is much more difficult to achieve uniform distribution of a large number of inorganic materials in the polymer matrix than in the laboratory scale. The cost is also a very important factor to consider when going out to synthetic route. The unprecedented surge in energy costs has made the regional polymer market unstable, which ultimately affects the cost of LMBs.

#### 4.2.2 Maturity and Scalability

In fact, the final factor affecting the application of ultrathin SPE is whether the fabrication technology is mature. Thus, in the manufacturing industry, a handbook is required to guide the reproducible and reliable fabrication of the ultrathin SPE. It is also important to transplant established polymer synthetic chemistries to the ultrathin SPE and will ultimately benefit the construction of various composite ultrathin SPE. Besides, for actual ultrathin SPE, operability/scalability is also a factor worth considering in practical mass manufacturing. Thus, scientists and technicians are encouraged to develop new concepts and methods for synthesizing ultrathin SPE, especially composite SPE and functionalizing its physicochemical properties. We emphasize the adjustment of reaction parameters as a very important research direction. Unfortunately, to our knowledge, little has been carried out in this area.

## 5 Fundamental Requirements and Design Principles for Future Ultrathin SPE

**Figure 5:**
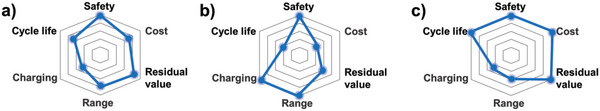
Consumer's choice for battery. a) Economical and practical; b) high‐performance; c) travel service. Battery manufacturing needs to be based on product positioning and customer demands.

From the perspective of the market, we classify the selection of LIBs according to the needs of consumers as follows (**Figure** **5**): a) economical and practical; b) high‐end performance; and c) travel service. Different demands have different requirements on the energy density, cost and charging/discharging rate of the LIBs. Thus, although solid‐state LMBs (such as: Li–S, Li‐NCM, et al.) based on ultrathin SPE show attractive prospects in energy density and safety, they also put forward higher requirements for ultrathin SPE.

### 5.1 Mechanical Strength and Ionic Conductivity

For most SPEs, ionic conductivity at room temperature and mechanical strength are often contradictory, that is, SPEs with high mechanical strength usually have low ionic conductivity. Therefore, how to decouple the ionic conductivity and mechanical properties of SPE is extremely important to promote the practical application of all solid‐state LMBs. For ultrathin SPE, ionic conductivity is not a big challenge, but mechanical strength is. For designing an ultrathin SPE with high mechanical strength, it is necessary to accurately control the polymerization of molecules and improve the degree of crosslinking while taking into account the flexibility of polymers, from the perspective of molecular design and synthetic chemistry. In this respect, our group designed a thiol‐branched SPE by cross‐linking the surface‐modified MOFs, PEGDA, and tetrakis (3‐mercaptopropionic acid) PETMP via —C—S—C bonds, obtaining an advanced SPE with loose interpenetrating network architecture, which demonstrated high Li^+^ conductivity (2.26 × 10^−4^ S cm^−1^ at room temperature) and good mechanical strength (9.4 MPa)/toughness (≈500%), thus unblocking the tradeoff between ionic conductivity and mechanical robustness in SPE. This work offers a new avenue to balance the mechanical strength and ionic conductivity and may reshape the molecular design of a polymer.

### 5.2 Electrochemical Window and Surface/Interface

There are two main challenges during designing LMBs, means: the significant volume change at the electrode/battery level and the unnecessary migration of cathode materials to anode materials.^[^ ^7^, ^34^ ^]^ For ultrathin SPE, the interface stability and narrow electrochemical window are two huge challenges because of its poor mechanical strength and high reactivity with Li metal, for which it is very easy to make the thin SPE, become thinner by dendritic penetration or reaction (corrosion), resulting in safety hazards. However, existing strategies: using inorganic solid‐state electrolytes with wide electrochemical windows as fillers, improving the unstable terminal group of PEO‐based polymer, et al.; do not increase the dispersion of inorganic materials in polymer matrix or the crosslinking degree between electrolytes. In some cases, due to the structural/chemical defects and charge redistribution, a large number of electrons/holes can form at the interface, leading to local decomposition of SPE and side reactions at the interface. Thus, for further ultrathin SPE design, while increasing the degree of crosslinking as much as possible to improve the electrochemical window, it is also necessary to consider/design the functional groups exposed on its surface, thereby ensuring closer chemical bonding with Li metal surface.^[^ ^35^ ^]^ For example, by modifying the surface functional groups of inorganic fillers, and then introducing them into the PEO‐based polymers with terminal modification by chemical crosslinking under the intermediate “bridge molecules”, achieving the uniform dispersion of inorganic fillers, thus, the electrochemical stability and the continuous ion transport channels can be synergistically realized.

### 5.3 Cost and Safety

Generally speaking, the cost of SPE is controllable and compatible with the current process level, but the current price of polymer batteries in the market is generally higher than that of LIBs, which affects their market capacities, and their ratio is about 1:9. Furthermore, ultrathin SPE may cause cost increase due to its high requirements on material and structure designs. Besides, safety is also a challenge for ultrathin SPE due to the unstable electrode/electrolyte interfaces. The current strategy is adding a small amount of liquid electrolyte to play the role of interface wetting, improving interface compatibility, however, it increases the safety hazards conversely. Based on this, we hold the opinion that maybe some phosphate groups can be introduced into ultrathin SPE from the viewpoint of safety.

## 6 Current Progress of Ultrathin SPE: Based on Synthetic Chemistry

### 6.1 Typical Polymerization Strategies for Obtaining Ultrathin SPE

#### 6.1.1 Photopolymerization

**Figure 6:**
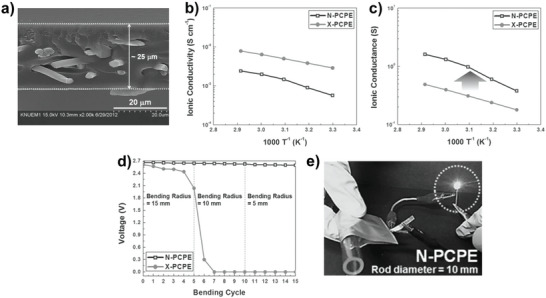
Photopolymerization and hydrosilylation for preparing ultrathin SPE. a) SEM image of the cross section of N‐PCPE. Reproduced with permission.^[^ ^37^ ^]^ Copyright 2014 Wiley‐VCH. b,c) Ionic conductivity (b) and ionic conductance (c) of N‐PCPE and X‐PCPE at given temperatures. Reproduced with permission.^[^ ^37^ ^]^ Copyright 2014 Wiley‐VCH. d,e) Comparison of cell voltage stability of cells using N‐PCPE and X‐PCPE under different deformation states. Reproduced with permission.^[^ ^37^ ^]^ Copyright 2014 Wiley‐VCH.

Now, photopolymerization is a typical strategy for the rapid preparation of high‐performance polymer electrolytes and has received a great deal of attention from researchers.^[^ ^36^ ^]^ To obtain a thin SPE (≈25 µm, **Figure** **6**a), ethoxylated trimethylolpropane triacrylate monomer was crosslinked by in situ UV‐crosslinking to form a 3D mesh plastic crystal polymer electrolyte matrix directly within the PET nonwoven fiber backbone in the co‐presence of plastic crystal electrolyte.^[^ ^37^ ^]^ The PET nonwoven is used as a flexible backbone to improve the mechanical strength/dimensional strength of the thin SPE. Although the ionic conductivity of this ultrathin electrolyte (named as N‐PCPE) is lower than that of X‐PCPE (without the PET nonwoven skeleton, Figure 6b), the ionic conductance of N‐PCPE film is significantly higher than that of X‐PCPE due to the thinness of N‐PCPE film and the shortened ion transport path (Figure 6c). Furthermore, thanks to such a unique structure, the obtained SPE significantly reduced film thickness and deformability, while maintaining its advantageous properties. Notably, the LMBs based on this thin SPE displayed stable electrochemical performance and no internal short‐circuit even in a severely crumpled state (Figure 6d,e).

#### 6.1.2 Hydrosilylation

**Figure 7:**
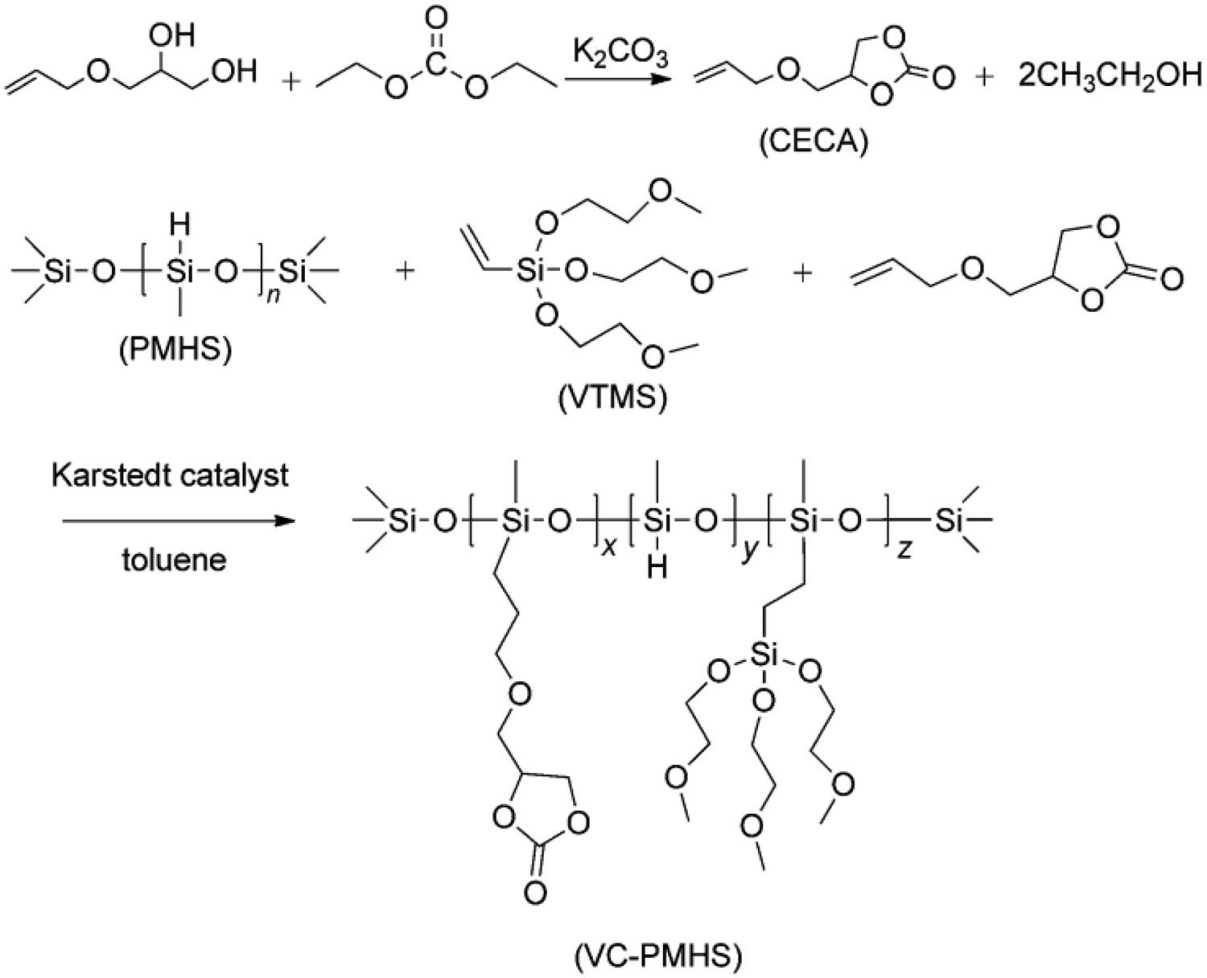
Synthetic route of polysiloxane. Reproduced with permission.^[^ ^38^ ^]^ Copyright 2014 Wiley‐VCH.

As a novel polymer matrix, polysiloxane was synthesized via hydrosilylation of polymethylhydrosiloxane with vinyl tris(2‐methoxyethoxy)silane and cyclic [(allyloxy)methyl]ethylene ester carbonic acid. A corresponding SPE film with thinness of 80 µm can be obtained (**Figure** **7**). However, the ionic conductivity at room temperature is not high enough (1.55 × 10^−4^ S cm^−1^), means: the LMBs with such a SPE need to be further improved or only used at high temperature (100 °C), which probably can be attributed to the nonconductive polysiloxane skeleton.^[^ ^38^ ^]^

#### 6.1.3 Ring Opening Polymerization

**Figure 8:**
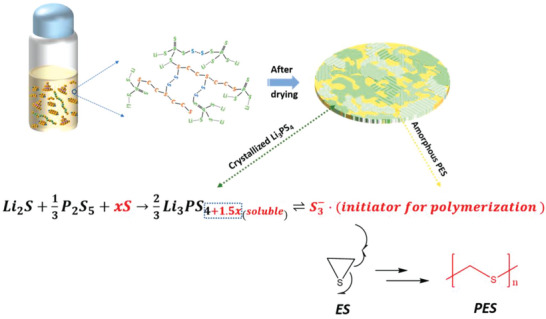
Synthesis and structure of the ultrathin SPE. Reproduced with permission.^[^ ^39^ ^]^ Copyright 2020 American Chemical Society.

Ring opening polymerization is also a common method to prepare thin SPE. A typical work was reported by Li et al., where they used a solvent synthesis method for exploring the potential chemical interactions between inorganic materials and polymers by the in situ ring opening polymerization (60 µm, **Figure** **8**).^[^ ^39^ ^]^ The authors choose *β*‐Li_3_PS_4_ as the inorganic filler because of its high ionic conductivity and simultaneously polymerized polyethylene sulfide (PES) in situ in this SPE, thereby bonding with *β*‐Li_3_PS_4_ through the polysulfide phase. There is no doubt that this strategy is highly innovative, and it provides a new idea for designing ultrathin SPE based on chemical synthesis, but there is still a broad research space to improve the ionic conductivity (2 × 10^−5^ S cm^−1^) at room temperature, meanwhile, cycling stability of the ultrathin SPE should also be given sufficient attention.

#### 6.1.4 Atom Transfer Radical Polymerization (ATRP)

**Figure 9:**
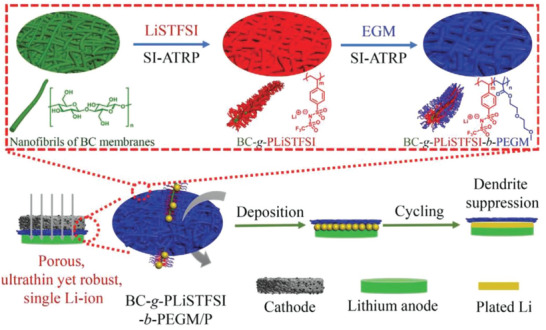
Atom transfer radical polymerization for preparing ultrathin SPE. Diagram of the structure and Li stripping/plating behavior of a SLIC‐QSPBE‐based Li metal battery. Reproduced with permission.^[^ ^40^ ^]^ Copyright 2021 Wiley‐VCH.

**Figure 10:**
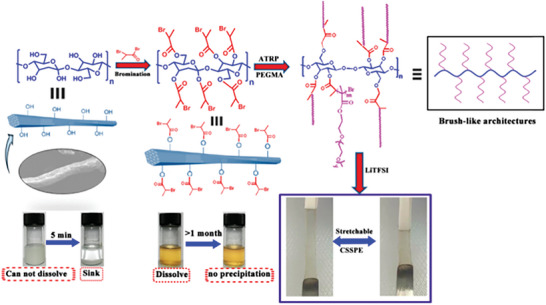
Synthetic route of bush‐like cellulose‐based electrolyte matrix. Reproduced with permission.^[^ ^41^ ^]^ Copyright 2020 American Chemical Society.

Inspired by the structure of both soft and hard tube brushes, a new super‐structured polymer brush bacterial cellulose‐*g*‐poly(4‐stylsulfonyl‐(trifluoromethylsulfonyl)imide lithium)‐*b*‐poly(diethylene glycol monomethyl ether methacrylate) with a hard nanofiber backbone and soft functional polymer side chains were synthesized via ATRP, which could well balance the mechanical strength and ionic conductivity (**Figure** **9**). The obtained SPE (named: SLIC‐QSPBEs) demonstrated an ultrathin film thickness (≈10 µm), a nanofiber backbone reinforced porous nanonetwork (Young's modulus = 1.9 GPa), and single ion‐conducting properties.^[^ ^40^ ^]^ As a result, the ultrathin and robust SLIC‐QSPBEs exhibited long‐term (over 3300 h) reversible and stable Li plating/stripping at a current density of 1.0 mA cm^−2^. Similarly, we also constructed a brush‐loaded polymer electrolyte matrix by grafting PPEMEA chain segments on cellulose via ATRP,^[^ ^41^ ^]^ and the obtained SPE can endow the corresponding LMBs to run stably for >450 cycles (**Figure** **10**). In fact, ATRP is a controlled polymerization strategy beyond traditional free radical polymerization, which can achieve well‐designed polymers with controllable molecular weight. Unfortunately, the complex synthetic route limits its further application.

As mentioned above, the extreme molecular designability of polymeric materials stems from their diverse synthesis methods, which bring unlimited possibilities for the design of high‐performance ultrathin polymer electrolytes. However, pure polymer components, in addition to maintaining high ionic conductivity when preparing ultra‐thin electrolyte, high strength is also required, otherwise the devices prepared are easy to short circuit, resulting in shortened device life. Therefore, in the future research of ultrathin polymer electrolytes with high strength and high ionic conductivity, the introduction of rigid substrates containing conductive ion transport channels should be increased, such as: the introduction of porous polymers, covalent organic skeletons^[^ ^42^ ^]^ and other materials containing abundant rigid units to construct microscopic porous ion channels, and the introduction of abundant rigid liquid crystal substrates in the polymer system to construct microscopic ordered ion transport channels is also promising.^[^ ^43^ ^]^ Based on these strategies, ultrathin electrolytes are expected to maintain high strength while not sacrificing ion transport efficiency.

### 6.2 Typical Composite Strategies for Obtaining Ultrathin SPE

#### 6.2.1 Layer by Layer Recombination

**Figure 11:**
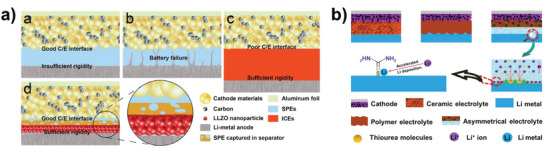
Layer by layer recombination for obtaining ultrathin SPE. a) Comparison of solid‐state Li metal cell with different electrolytes. Reproduced with permission.^[^ ^42^ ^]^ Copyright 2018 American Chemical Society. b) Schematic illustration of different electrolytes and mechanism of THU. Reproduced with permission.^[^ ^6c^ ^]^ Copyright 2020 Royal Society of Chemistry.

The key bottleneck plaguing SPE applications is the conflicting requirements of cathode and Li metal, requiring flexibility to ensure low interfacial resistance and high modulus to avoid dendritic Li penetration, respectively. Thus, Guo group developed a thin asymmetric solid electrolyte (below 36 µm) with engineered layers (**Figure** **11**a).^[^ ^42^ ^]^ Although the thin film shows unsatisfied ionic conductivity (10^−4^ S cm^−1^ at 55 °C), the interfacial resistance of the assembled cell is low (maybe because the as‐prepared electrolyte film is ultra‐thin, which can reduce the ionic conductive route). As a result, a dendrite‐free Li metal cell with long cycle life was successfully achieved. Similarly, our group also reported a thin asymmetric SPE (≈40 µm), where the gel polymer electrolyte (PVDF‐HFP) with thiourea is used on the anode side of the battery and a solid electrolyte PEO‐LLZO on the cathode side (Figure 11b).^[^ ^6c^ ^]^ The obtained composite SPE exhibited a high Li‐ion conductivity of 3.8 × 10^−4^ S cm^−1^ at 25 °C. And the symmetric Li|Li cell can maintain a stable Li plating/stripping process over 1600 h. Besides, solid LMBs showed stable cycling performances at 1.0 C over 200 cycles with ≥99.5% CE. Recently, Ma et al. prepared a high‐performance, ultra‐thin electrolyte (≈16 µm) by modifying the surface of the diaphragm, but the introduction of a large amount of liquid electrolyte sacrificed the safety of the battery.^[^ ^29^ ^]^ It is also because of this, the obtained SPE demonstrated a high Li‐ion conductivity of 7.8 × 10^−4^ S cm^−1^ at room temperature and high electrochemical stability of 5.25 V (versus Li/Li^±^). The full cells with LiNi_0.5_Mn_1.5_O_4_ cathode also displayed a superior cycling stability and rate performance at room temperature.

#### 6.2.2 Organic–Inorganic Hybrid

In fact, PEO is still the main component used in the preparation of ultra‐thin composite electrolytes. A thin‐layer composite solid electrolyte (LCSE), Vr/PEO‐LCSE (10 µm), was prepared by filtering vermiculite nanosheets and inserting PEO‐LiTFSI between the layers by means of swelling and filtration. Although the ionic conductivity of the composite electrolyte is low (1.22 × 10^−5^ S cm^−1^ at 25 °C), Li|Vr/PEO‐LCSE|S cell can cycled at 60 °C.^[^ ^41^ ^]^ Recently, Wang et al. developed an ultrathin hydrotalcite nanosheet‐promoted PVDF‐HFP‐based composite SPE (the detailed thickness is not provided in the reference) that required only ≈1 wt% inorganic filler to achieve a room‐temperature ionic conductivity of 2.2 × 10^−4^ S cm^−1^, a Li^+^ transfer number of ≈0.78, and an electrochemical stability window of 4.9 V. This work is the first to achieve stable cycling of PVDF‐HFP‐based all‐solid‐state LMBs at room temperature, providing a new strategy to promote the practical application of low‐cost, high‐performance composite SPE.^[^ ^43^ ^]^ Metal–organic framework with abundant nanochannels can promote the transport of Li^±^. Especially, the 1D fibrous structure of MOF can form 3D inter‐linked matrix via intertwined with each other, which can further increase the strength of composite electrolytes. Typically, Shi et al. prepared an ultra‐thin composite electrolyte (40 µm) by combining 1D Cu‐MOF and PVDF‐based electrolyte, thereby achieving high‐performance LMBs.^[^ ^44^ ^]^ Nevertheless, liquid electrolyte was introduced to the composite electrolyte, which sacrifices the mechanical strength and the safety of the membrane to some extent.

Compared with organic–inorganic hybrid, organic–organic hybrid can endow the composite electrolyte with better interface compatibility, a composite electrolyte based on a lithiophilic polyacenequinone polymer filler and thermoplastic polyurethane (polymer electrolyte matrix) was proposed by Ye et al. and this strategy ensured the composite electrolyte high mechanical flexibility, dendrite‐free Li deposition, and LMBs with high‐voltage cathode (NCM811) also exhibited stable cycle performance.^[^ ^45^ ^]^ However, because of the low conductivity of polymer filler and polymer electrolyte matrix, liquid electrolyte should be introduced.

#### 6.2.3 Composite Based on Porous Ceramic Framework

**Figure 12:**
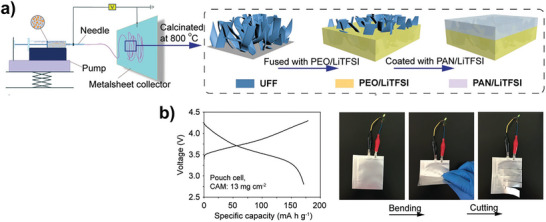
Composite ultrathin SPE based on porous ceramic framework. a) Preparation route of an ultra‐thin composite SPE. Reproduced with permission.^[^ ^46^ ^]^ Copyright 2021 Wiley‐VCH. b) Charging/discharging curves of the pouch cell using this ultra‐thin SPE, and corresponding pouch cell lights an LED under bending and cutting. Reproduced with permission.^[^ ^46^ ^]^ Copyright 2021 Wiley‐VCH.

The thickness of the SSE is a key parameter affecting the energy density. Theoretically, the energy density at the cell level is inversely proportional to the thickness of the SSE. Recently, He et al. proposed an ultrathin (4.2 µm) and lightweight (1.29 g cm^−3^) bilayer SPE with a 3D ultrathin framework embedded in a polymer electrolyte (**Figure** **12**).^[^ ^46^ ^]^ The ultrathin composite SPE combined the rigid ceramic filler and soft polymer electrolyte, where the ceramic filler enhanced the mechanical strength and the bilayer polymer electrolyte stabilized Li metal anode and the high‐voltage cathode. The increased ionic conductivity and Li^+^ migration number facilitated the regulated Li deposition and improved CE. The pouch cells with cathode active materials loading of 13 mg cm^−2^ at 0.45 mA cm^−2^ reached capacity of 174 mAh g^−1^ and can light an LED under bending and cutting states (Figure 12b). High energy densities of more than 500 Wh kg^−1^ and 1500 Wh L^−1^ were achieved for solid LMBs. The full cells ultimately achieved an extended life with a high average CE of 99.2%. It is expected that such an ultra‐thin SPE will provide an example and lead the way for high energy density LMBs in large‐scale energy storage systems. There is another interesting work, where a polytetrafluoroethylene (PTFE) binder interconnected LLZTO self‐supported 3D skeleton without any solvent was prepared by a simple grinding method.^[^ ^47^ ^]^ Subsequently, composite electrolytes were prepared by filling the flexible 3D LLZTO framework with a succinonitrile solid electrolyte. The full cells with NCM cathode assembled with this thin composite electrolyte exhibited a discharge specific capacity of 158 mAh g^−1^ and high cycling stability at room temperature. This work emphasizes that the electrolyte film is thin (100 µm), but the thickness is actually thicker compared to other works, and further reducing the thickness (while maintaining the strength) might will further enhance the performance of corresponding LMBs and devices. In addition, Zou et al. also reported a flexible, flame‐resistant and dendrite‐free composite electrolyte based on thermoplastic polyurethane and aerogel SiO_2_,^[^ ^48^ ^]^ it is hoped that the thickness (250 µm) of obtained composite electrolyte film can be further reduced, thereby improving the ionic transport ability and the energy density of corresponding LMBs.

#### 6.2.4 Composite Based on Porous Polymer Framework

**Figure 13:**
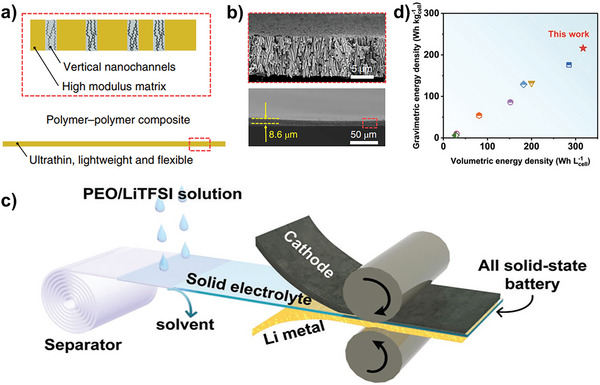
Composite ultrathin SPE based on porous polymer framework. a,b) Design principles of the ultra‐thin SPE and its SEM images. Reproduced with permission.^[^ ^30^ ^]^ Copyright 2019 Nature Publishing Group. c) Fabrication process of the ultra‐thin SPE. Reproduced with permission.^[^ ^49^ ^]^ Copyright 2019 Wiley‐VCH. d) Comparison of gravimetric and volumetric energy densities with references. Reproduced with permission.^[^ ^49^ ^]^ Copyright 2019 Wiley‐VCH.

**Figure 14:**
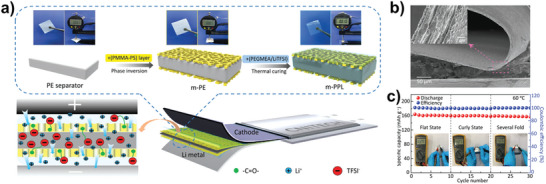
Composite ultrathin SPE based on porous separator framework. a) Preparation route of the ultra‐thin SPE. Reproduced with permission.^[^ ^50^ ^]^ Copyright 2021 Wiley‐VCH. b) SEM image of the ultra‐thin SPE under bending state. Reproduced with permission.^[^ ^47^ ^]^ Copyright 2021 Wiley‐VCH. c) The cycling ability under different deformation states. Reproduced with permission.^[^ ^50^ ^]^ Copyright 2021 Wiley‐VCH.

Besides, porous membrane materials with uniform thickness can be used as a backbone (e.g., PI, PE diaphragm) for filling, thereby obtaining composite SPE with uniform thickness. A typical example was reported by Cui group, where they prepared a nanoporous polyimide (PI) membranes filled with PEO/LTFSI (≈8 µm) for all‐solid‐state Li batteries (**Figure** **13**a,b).^[^ ^30^ ^]^ In this novel composite SPE, the vertically aligned nanochannels enhanced the ionic conductivity of PEO/LTFSI (2.3 × 10^−4^ S cm^−1^ at 30 °C), thus the obtained LMBs demonstrated excellent cycling performance and can withstand abuse tests such as bending, cutting and nail penetration. Unfortunately, the battery has to be operated at high temperatures (60 °C). But anyway, this work inspires researchers that the performance of the corresponding ultra‐thin electrolytes and devices can be further improved by designing other types of polymer electrolytes instead of PEO. Simultaneously, Huang group also reported an ultrathin flexible SPE with a thickness of only 7.5 µm by filling a polyethylene oxide (PEO)/lithium bis(trifluoromethanesulfonyl)imide (LiTFSI) polymer electrolyte into the pores of a polyethylene diaphragm using a simple solvent volatilization method (Figure 13c). Obviously, the gravimetric/volumetric energy densities can be strongly increased (Figure 13d, compared with references).^[^ ^49^ ^]^ Furthermore, for increasing the ionic conductivity further, there is an interesting ultrathin SPE, consisting of a modified polyethylene (PE) diaphragm as the main body and poly(ethylene glycol) methacrylate and Li salts as fillers (**Figure** **14**).^[^ ^50^ ^]^ The introduction of porous poly(methyl methacrylate)‐polystyrene interfacial layer can tightly attach to the PE sides effectively, improving the interfacial compatibility with electrode. Lastly, the 10 µm‐thick SPE has an ultra‐high ionic conductivity of 34.84 mS at room temperature, as well as a mechanical property of 103.0 MPa with an elongation of 142.3%. A corresponding symmetric cell can be stably cycled at 60 °C for more than 1500 h and pouch full cells with LiFePO_4_ can be run stably for more than 1000 cycles at 1 C with a capacity retention rate of 76.4%. What's more, it can still work stably after curling and folding, proving its excellent flexibility and safety.

**Figure 15:**
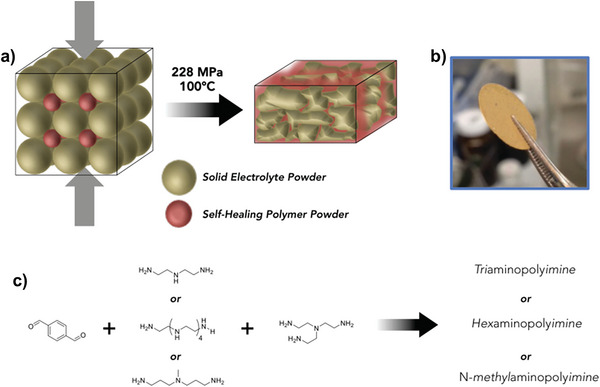
Preparation concept of a unique ultra‐thin SPE. Reproduced with permission.^[^ ^51^ ^]^ Copyright 2015Wiley‐VCH.

In addition to the polymer skeleton mentioned above, there is an interesting ultrathin SPE, which started with the block powder and took the advantage of the ductility of polyimide (a self‐healing material), thus, the hot isostatic press can form a continuous cross‐linked network between the voids of glass‐ceramic, increasing the density of composite SPE (**Figure** **15**).^[^ ^51^ ^]^ Thus, the obtained composite SPE can demonstrate a thickness of 63.7 µm. However, this self‐healing material can dramatically reduce the ionic conductivity of composite SPE, which may be the reason for poor electrochemical performance. Subsequently, in future research, it may be possible to further design self‐healing polymers with high ionic conductivity to composite the corresponding inorganic fillers, thereby enhancing various properties of composite ultrathin SPE.

Compared with pure ultra‐thin polymer electrolytes, the mainstream strategy is to compound different types of materials with polymer electrolytes for preparing ultra‐thin composite electrolytes, including inorganic/organic nanoparticle fillers, inorganic nanowire fillers and inorganic/organic 3D skeletal materials. Inorganic nanoparticle fillers can significantly improve the strength of ultrathin composite electrolytes and inhibit the crystallization of polymers, but their homogeneous composite is a great challenge, especially the agglomeration of inorganic nanoparticle fillers may lead to the reduction of the local strength of ultrathin composite electrolytes. Organic nanoparticles and polymer electrolytes have better compatibility compared to inorganic nanoparticle fillers, but the strength of their composites is an issue. Compared with inorganic particulate nanofillers, inorganic nanowires can not only improve the strength of polymer electrolytes and inhibit the crystallization of polymer electrolytes, but also the interface formed by their nanowires and polymers can further promote ion transport, however, the preparation of uniform ultra‐thin composite electrolytes still needs further exploration. Inorganic/organic 3D skeleton materials have unique advantages over nanoparticle and nanowire fillers and are an important direction for the future development of high‐performance ultrathin electrolytes. This is because the 3D skeleton structure can be prepared in advance as an ultrathin electrolyte support matrix with high strength and controllable thickness. Based on this, compounding with lower molecular weight polymer electrolyte can make the ultra‐thin electrolyte take into account the high strength of 3D skeleton and high ionic conductivity of low molecular weight polymer electrolyte, and it is expected to solve the interface problem between the composite electrolytes. However, it is still necessary to further improve the electrochemical stability of the polymer electrolyte by molecular design in order to better match with Li metal anode and high‐voltage cathodes.

## 7 Summary and Outlook

As a “Holy Grail” anode, Li metal anode has been regarded as a new generation of high specific energy anode material with great potential. However, the side reaction between Li anode and electrolyte is very severe, which seriously restricts its further commercial application. Developing high‐performance SPE is not only an important means to solve the above challenges, but also a current research hotspot. During the development and research of SPE, ultrathin SPE gradually came into the researchers' view, and quickly attracted extensive attention. Although we have witnessed great success to this day, we still face great challenges. In this section, we discuss the existing challenges and future opportunities of ultrathin SPE. Our goal is to provide the readers with critical and in‐depth evaluation of the current progress and future possibilities of advanced SPE design. An overview is given from the merits and applications of ultrathin SPE, to its existing challenges and design principles. Then, we innovatively summarized and discussed the current progress of ultrathin SPE from the perspective of synthetic chemistry, including: various means of polymerization and diversified compound strategies. Specifically, it is summarized as follows:

For polymerization, various synthesize strategies such as photopolymerization, hydrosilylation, ring opening polymerization, and ATRP have been adopted to construct ultrathin SPE. Photopolymerization is the most widely used method because it has the advantages of high efficiency, simple synthesis process, and easy batch preparation. Hydrosilylation is a recently reported method for constructing ultra‐thin SPE, mainly by improving the mechanical properties of electrolytes with the help of high elasticity of silicone rubber materials, which should provide a new method for the design and preparation of ultra‐thin SPE with excellent mechanical properties, but the insulating characteristics of silicon‐based materials bring new challenges for efficient ion transport of ultra‐thin SPE. Similar to photopolymerization, ring‐opening polymerization usually does not require the introduction of additional solvents, but only requires the selection of suitable monomers and the addition of initiators and lithium salts to allow the monomers to react to obtain a suitable electrolyte. However, the usually limited selectivity of ring‐opening polymerization monomers and the uncontrollable molecular weight of the polymers are not conducive to purposeful tuning of the polymer properties. ATRP is a typical reactive controlled radical polymerization method, which not only has the advantage of wide selection of polymerization monomer types, but also has controllable polymer molecular weight, providing the possibility of purposeful design of high‐performance polymer electrolyte matrix, but ATRP has the disadvantages of harsh reaction conditions and difficulties in product separation and purification.

For composite, the strategy of compounding can give two or more components of ultrathin electrolytes their respective advantages, that is, achieve the effect of 1 + 1 is greater than 2. For ultra‐thin electrolytes, the composite strategy has its unique advantages. Representatively, a high‐strength, uniform‐thickness component is used as the backbone (e.g., diaphragm, ceramic membrane, polyimide membrane, etc.), and the polymer electrolyte prepared by chemical synthesis is poured on the backbone to obtain a uniform‐thickness, high‐strength ultra‐thin electrolyte.

Since the development of ultrathin SPE is still at an infantile stage, there remain many issues that need to be solved to promote their practical applications. We here highlight several typical challenges and perspectives in the field. In likewise, we mainly focus on synthetic chemistry.

### 7.1 Thickness‐Property Relationship

Disclosing the regulating effect of thickness on the basic attributes of ultrathin SPE can in return promote their design and applications. Opportunities exist in tuning/optimizing the thickness of ultrathin SPE to its mechanical strength, ionic conductivity, flexibility, electrochemical stability, structural stability, safety, et al., and exploring novel properties and device applications based on integrating these interplays.

The first topic focuses on the tradeoff of thickness and mechanical strength. There is no doubt that reducing the thickness has a great impact on the mechanical strength. In that case, the mechanical strength can only be improved by increasing the degree of crosslinking of the polymers, unfortunately, this strategy will lead to an increase in polymer crystallinity, reducing ionic conductivity. Although our group has proposed to introduce intermediate “bridge molecules” to increase the degree of polymer crosslinking while ensuring polymer slip, more follow‐up is needed. Another topic is the thickness and stability. When the thickness of ultrathin SPE is reduced to a certain extent, the deformation degree of the ultrathin SPE increases with the dendritic Li growth, resulting in drastic fluctuations in the structural stability of ultrathin SPE and the interface stability, thereby affecting the cycling stability of LMBs. It is particularly important for ultrathin SPE to regulate the deposition behavior of Li metal. For example, our group constructed an asymmetric ultrathin SPE. In the quasi‐solid electrolyte toward anode, trace thiourea molecules can effectively regulate the deposition behavior of Li metal, avoid the formation of Li dendrites, and thus ensuring that the ultrathin SPE will hardly deform during the charging/discharging process. In all, design and innovation at the molecular level is an important research direction of ultrathin SPE, which may direct the research in the near future.

### 7.2 Synthesis and Analysis

**Figure 16:**
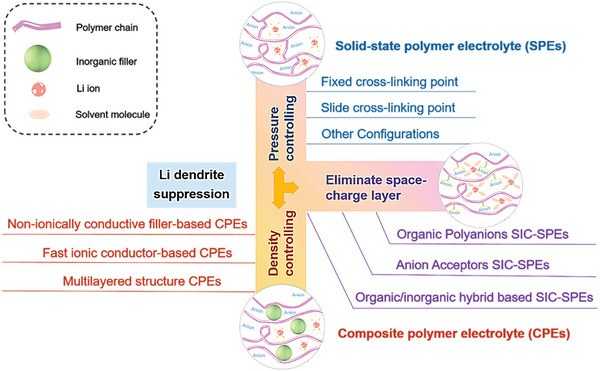
Diversified SPE types under thick scale. SIC means: single ion conductor; CPEs means: composite polymer electrolytes.

Now, for thicker SPE, researchers have used a large number of means, such as: molecular design (copolymerization, crosslinking, grafting, etc.), composite (blending, organic–inorganic composite), and polymer alloying to inhibit polymer crystallization and improve the creepage of the polymer chains. Various SPE are thus derived, such as: single‐ion conducting SPE, composite SPE, et al. (**Figure** **16**). However, for ultrathin SPE, perhaps for the sake of mechanical strength, there are few publications related to the current research. In further development, more attempts and explorations on synthesis methods are needed to be explored to realize the transition of SPE from thick to thin, especially in the SPE with unique properties, such as: single‐ion conducting SPE. Besides, for composite SPE, organic–inorganic composite can not only improve the ionic conductivity of SPE to a certain extent, but also comprehensively improve its interface performance, electrochemical stability and mechanical properties, which have attracted much attention in recent years. However, how to ensure the uniform dispersion of inorganic materials and its stability in the battery cycle (not “overflow” from the SPE) needs further research.

For analyzing ultrathin SPE, the current research on the transport characteristics of Li^+^ lags behind, especially the transport path of Li^+^ at the two‐phase interface after organic–inorganic recombination. We believe that the relationship between the geometric characteristics, physical and chemical properties of inorganic fillers and the Li^+^ transport characteristics of Li^+^ will be explored by combining X‐ray diffraction (XRD), differential thermal analysis (TG‐DSC), infrared spectroscopy (IR), micro electrochemical system, freezing electron microscopy, et al., while the structure–activity relationship between structure and performance will be revealed. Besides, developing advanced and high‐resolution surface/interface detection technology at 3D level is of great significance to deeply understand the surface/interface chemical behavior and dynamic characteristics of ultrathin SPE, thereby guiding its subsequent design.

### 7.3 Application

In fact, the final factor affecting the application of ultrathin SPE is whether the fabrication technology is mature. Through in‐depth study of the relationship between thickness/materials and performance, conducting data statistics, and summarizing the relevant laws. Thus, in the manufacturing industry, a handbook is required to guide the reproducible and reliable fabrication of the ultrathin SPE. In the technical route, scientists and technicians are encouraged to develop new concepts and methods for synthesizing ultrathin SPE or composite ultrathin SPE and functionalizing their surface, thereby ensuring high interface stability. However, it is rarely reported due to the complexity in surface interactions.

## 8 Conclusion

In summary, ultrathin SPE represent a novel direction having different new knowledge from conventional SPE. However, the huge challenges in balancing the properties of all aspects limit its further application, but also imply great opportunities in developing innovative structures and functionalities. In this review, we discussed the advantages and existing challenges of ultrathin SPE, including scientific problems and technical bottlenecks. Meantime, we summarized the progress of ultrathin SPE based on synthetic chemistry and looked back upon several typical fabrication methods. We cover all existing cases related to this field as much as possible, and hope to provide several examples that reflect the trends and possible opportunities in designing ultrathin SPE in the future. Enabled by a deep insight into the synthetic chemistry of ultrathin SPE, there are tremendous prospects in advanced LMBs.

## Conflict of Interest

The authors declare no conflict of interest.
